# Rifampicin-Induced Hepatic Lipid Accumulation: Association with Up-Regulation of Peroxisome Proliferator-Activated Receptor γ in Mouse Liver

**DOI:** 10.1371/journal.pone.0165787

**Published:** 2016-11-02

**Authors:** Jia-Hui Huang, Cheng Zhang, Da-Gang Zhang, Lu Li, Xi Chen, De-Xiang Xu

**Affiliations:** 1 First Affiliated Hospital, Anhui Medical University, Hefei, China; 2 Department of Toxicology, Anhui Medical University, Hefei, China; University of Basque Country, SPAIN

## Abstract

Previous study found that rifampicin caused intrahepatic cholestasis. This study investigated the effects of rifampicin on hepatic lipid metabolism. Mice were orally administered with rifampicin (200 mg/kg) daily for different periods. Results showed that serum TG level was progressively reduced after a short elevation. By contrast, hepatic TG content was markedly increased in rifampicin-treated mice. An obvious hepatic lipid accumulation, as determined by Oil Red O staining, was observed in mice treated with rifampicin for more than one week. Moreover, mRNA levels of *Fas*, *Acc* and *Scd-1*, several key genes for fatty acid synthesis, were elevated in rifampicin-treated mice. In addition, the class B scavenger receptor CD36 was progressively up-regulated by rifampicin. Interestingly, hepatic SREBP-1c and LXR-α, two important transcription factors that regulate genes for hepatic fatty acid synthesis, were not activated by rifampicin. Instead, hepatic PXR was rapidly activated in rifampicin-treated mice. Hepatic PPARγ, a downstream target of PXR, was transcriptionally up-regulated. Taken together, the increased hepatic lipid synthesis and uptake of fatty acids from circulation into liver jointly contribute to rifampicin-induced hepatic lipid accumulation. The increased uptake of fatty acids from circulation into liver might be partially attributed to rifampicin-induced up-regulation of PPARγ and its target genes.

## Introduction

Nonalcoholic fatty liver disease (NAFLD) has been considered the most common chronic liver disease in developed countries and has been gradually increasing in Chinese adults in recent decades [1,2]. The hallmark of NAFLD is excessive triglyceride (TG) accumulation in liver [3]. NAFLD represents a wide spectrum liver disease ranging from simple hepatic steatosis to nonalcoholic steatohepatitis, fibrosis, cirrhosis, and even hepatocellular carcinoma [4]. Although obesity, type 2 diabetes, high-fat diets, insulin resistance and metabolic syndrome have been recognized as common risk factors of NAFLD [5], drug-induced hepatic steatosis and steatohepatitis are considered a rare but very important forms of NAFLD [6].

Rifampicin, one of the most commonly used front-line drugs in antituberculosis therapy, has been well known to be hepatotoxic [7, 8]. Two in vitro studies showed that rifampicin caused a direct toxic injury to rat hepatocytes [9, 10]. Several in vivo studies found that rifampicin plus isoniazid induced hepatocyte apoptosis in rodent animals [11–13]. The mechanism through which rifampicin induces liver injury remains obscure. An earlier study demonstrates that oxidative stress in the mitochondria is involved in the pathogenesis of rifampicin plus isoniazid-induced apoptotic liver cell injury in mice [14]. According to a report from our laboratory, rifampicin causes intrahepatic cholestasis through altering integrity of hepatocyte ZO-1 and occluding [15].

The aim of the present study was to explore the effects of rifampicin on hepatic lipid metabolism in mice. We showed that rifampicin elevated hepatic TG content and caused hepatic lipid accumulation. We found that rifampicin up-regulated genes for synthesis and transport of hepatic fatty acids. Our results suggest that the increased hepatic lipid synthesis and uptake of fatty acids from circulation into liver partially contribute to rifampicin-induced hepatic lipid accumulation. The increased uptake of fatty acids from circulation into liver might be attributed to rifampicin-induced up-regulation of peroxisome proliferator-activated receptor γ (PPARγ) and its target genes.

## Materials and Methods

### Chemicals

Rifampicin was purchased from Sigma Chemicals Co. (St. Louis, MO, USA). Antibodies against SREBP-1c, LXR-α, PXR, PPARγ, and Lamin A/C were from Santa Cruz Biotechnologies (Santa Cruz, CA, USA). Chemiluminescence (ECL) detection kit was from Pierce Biotechnology (Rockford, IL, USA). TRI reagent was from Invitrogen (Carlsbad, CA, USA). RNase-free DNase was from Promega Corporation (Madison, WI, USA). Oil Red O was from Sigma Chemical Co. (St Louis, MO, USA). All the other regents were from Sigma or as indicated in the specific methods.

### Animals and treatment

Thirty-two male CD-1 mice were purchased from the Laboratory Animal Center of Anhui Medical University. The animals were maintained on a 12-h light/dark cycle in a controlled temperature (20–25°C) and humidity (50±5%) environment for a period of 1 week before use. All mice were fed with regular diet. To investigate the effect of rifampicin on hepatic lipid accumulation, mice were divided into four groups. All mice except controls were orally administered with rifampicin (200 mg/kg) daily for 3 days, 1 week or 4 weeks, respectively. All thirty-two male CD-1 mice survived to the end of the experiments. All mice were anesthetized with ether and then sacrificed with dislocation of cervical vertebrae 6 h after the last rifampicin treatment. Serum was collected for measurement of biochemical parameters. Liver was collected and frozen immediately in liquid nitrogen for hepatic TG measurement, real-time RT-PCR and Western Blot, fixed in neutral-buffered formalin for histological examination, or frozen fixed in OCT mounting media for Oil red O staining. This study was approved by the Association of Laboratory Animal Sciences and the Center for Laboratory Animal Sciences at Anhui Medical University (Permit Number: 15–0010). All procedures on animals followed the guidelines for humane treatment set by the Association of Laboratory Animal Sciences and the Center for Laboratory Animal Sciences at Anhui Medical University. In this study, all mice were monitored at least twice per day. In addition, the rules of humane endpoints were strictly performed to determine when mice should be euthanized. All efforts were taken to minimize suffering when mice met our euthanasia criteria.

### Biochemical parameters and hepatic histology

Serum alanine transaminase (ALT), TG, total cholesterol (TC), high density lipoprotein cholesterol (Chol-HDL) and very low density lipoprotein TG (TG-VLDL) were measured by routine laboratory methods using an autoanalyzer (Roche, Modular DPP, NO. 1549–06). Liver specimen was fixed in 4% paraformaldehyde phosphate buffer. Liver sections were stained with hematoxylin and eosin and evaluated by the pathologists who were blind to sample assignment to experimental groups.

### Hepatic TG measurement

For measurement of hepatic TG content, liver samples were homogenized in 2 ml of buffer that contains 18 mM Tris (pH 7.5), 300 mM mannitol, 50 mM EGTA, and 0.1 mM PMSF. For extraction of hepatic TG, 400 μl of tissue homogenate was mixed with 4 ml of chloroform/methanol (2:1) and incubated with shaking overnight at room temperature. Finally, 800 μl of H_2_O was then added and vortexed, centrifuged at 3000 g for 5 min, and the lower lipid phase was collected and dried. The lipid pellets were then dissolved in a 100 μl mixture of tert-butyl alcohol/ Triton X-114/methanol (4.6:2:1) [16]. TG was determined by a commercially available kit. Hepatic TG contents were expressed as μmol/g liver.

### Oil Red O staining

To determine hepatic lipid accumulation, frozen sections of liver (10 μm) were stained with Oil Red O for 10 min, washed, and counterstained with hematoxylin for 45s. Representative photomicrographs were captured at 100× magnification using a system incorporated in the microscope.

### Isolation of total RNA and real-time RT-PCR

Total RNA was extracted using TRI reagent. RNase-free DNase-treated total RNA (1.0 μg) was reverse-transcribed with AMV (Promega Corp., Madison, WI, USA). Real-time RT-PCR was performed with Light Cycler 480 SYBR Green I Kit (Roche Diagnostics GmbH, Manheim, Germany) using genetic-specific primers, as listed in Table 1. The amplification reactions were carried out on a Light Cycler 480 Instrument (Roche Diagnostics GmbH, Mannheim, Germany) with an initial hold step (95°C for 5 minutes) and 50 cycles of a three-step PCR (95°C for 15 seconds, 60°C for 15 seconds, 72°C for 30 seconds). The comparative CT-method [17] was used to determine the amount of target, normalized to an endogenous reference (*18S*) and relative to a calibrator (2-ΔΔCt) using the LightCycler 480 software (Roche, version 1.5.0).

**Table 1:** Oligonucleotide sequence of primers for RT–PCR.

Genes	Forward (5’-3’)	Reverse (5’-3’)
*18s*	GTA ACC CGT TGA ACC CCA TT	CCA TCC AAT CGG TAG TAG CG
*Fas*	TAC TTT GTG GCC TTC TCC TCT GTA A	CTT CCA CAC CCA TGA GCG AGT CCA GGC CGA
*Acc*	CCG TTG GCC AAA ACT CTG GAG CTA A	GAG CTG ACG GAG GCT GGT GAC A
*Scd-1*	GCC AGA CCG GGC TGA ACA CC	GGC CTC CCA AGT GCA GCA GG
*Cpt-1α*	TTC CCC GCG AGT CCC TCC AG	TGG GCC AGT GCT GTC ATG CG
*Cyp4a10*	TAT GTG AAA AAC ATG GCC GA	TCT TTT CCA GCT CTC CCT CA
*Cyp4a14*	GAT GTT GAC TCC AGC CTT CC	CAT TCT GCA GCT GAG ACT TCC
*Cd36*	GAT GAC GTG GCA AAG AAC AG	AAA GGA GGC TGC GTC TGT G
*Fatp*	CGC CGA TGT GCT CTA TGA CT	ACA CAG TCA TCC CAG AAG CG
*L-fabp*	GGA AGG ACA TCA AGG GGG TG	TCA CCT TCC AGC TTG ACG AC
*Mttp*	GTT TTT CCC GGT CAA GCG TT	TTT CAG TGG GGC GAT CTT CG
*Apob*	AAG ACC ATC CTG AGC CAG AC	TTA TGC CAG CTT GGT TGC AG
*Ldlr*	TCA CAC AGC CTA GAG AAG TCG	ATC CTC ACT GTG CTT CGG TG
*Pparγ*	GGG CTG AGG AGA AGT CAC AC	TCA GTG GTT CAC CGC TTC TT
*Cyp3a11*	CCT GGG TGC TCC TAG CAA TC	GGC CCA GGA ATT CCC TGT TT

### Nuclear protein extraction

For nuclear protein extraction from the liver, 400 mg liver tissue was homogenized in 5 mL ice-cold buffer A [10 mM HEPES (pH 7.9), 150 mM NaCl, 0.6% NP-40, 0.1 mM EDTA, 1mM dithiothreitol (DDT), and 0.5 mM phenylmethylsulfonyl fluoride (PMSF)] on ice. The homogenate was centrifuged at 270× g for 30 s and the precipitate was discarded. The supernatant was kept on ice for 5 min and centrifuged again at 3,000× g for 20 min at 4°C. The supernatant was then mixed with 1 mL ice-cold buffer A and centrifuged again at 3, 000× g for 5 min. The precipitate containing nuclei was reserved and homogenized in 100 μL Buffer B [20 mM HEPES (pH 7.9), 420 mM NaCl, 1.2 mM MgCl2, 25% glycerol, 0.2 mM EDTA, 0.5 mM DDT, 0.5 mM PMSF, 1% protease inhbitor cocktail (P8340, Sigma)] for 60 min on ice. Nuclear lysate was centrifuged at 11, 000× g for 10 min at 4°C. The supernatant was collected and protein concentrations were determined with the bicinchoninic acid (BCA) protein assay reagents (Pierce, Rockford, IL, USA) according to the manufacturer’s instructions. For nuclear protein extraction from cells, the cells were washed with ice-cold PBS/phosphatase inhibitors, collected with a cell scraper, and harvested by centrifugation. The cell pellet was then resuspended in hypotonic buffer and then kept on ice for 15 min. The suspension was then mixed with detergent and centrifuged for 30 s at 14, 000× g. The nuclear pellet obtained was resuspended in complete lysis buffer in the presence of the protease inhibitor cocktail, incubated for 30 min on ice, and centrifuged for 10 min at 14,000 × g. Protein concentrations were determined with the BCA protein assay reagents.

### Western blot

Nuclear extracts were separated electrophoretically by SDS-PAGE and transferred to a polyvinylidene fluoride membrane. The membranes were incubated for 2 h with the following antibodies: SREBP-1, LXRα, PXR and PPARγ. Lamin A/C was used as a loading control for nuclear proteins. After washed in DPBS containing 0.05% Tween-20 four times for 10 min each, the membranes were incubated with goat anti-rabbit IgG antibody for 2 h. The membranes were then washed in DPBS containing 0.05% Tween-20 for four times for 10 min each again, followed by signal development using an ECL detection kit.

### Statistical analysis

All data were expressed as means ± SEM. SPSS 13.0 statistical software was used for statistical analysis. All statistical tests were two-sided using an alpha level of 0.05. ANOVA and the Student-Newmann-Keuls post hoc test were used to determine differences among different groups. Student *t* test was used to determine differences between two groups.

## Results

### Rifampicin induces hepatic lipid accumulation

**Fig 1:**
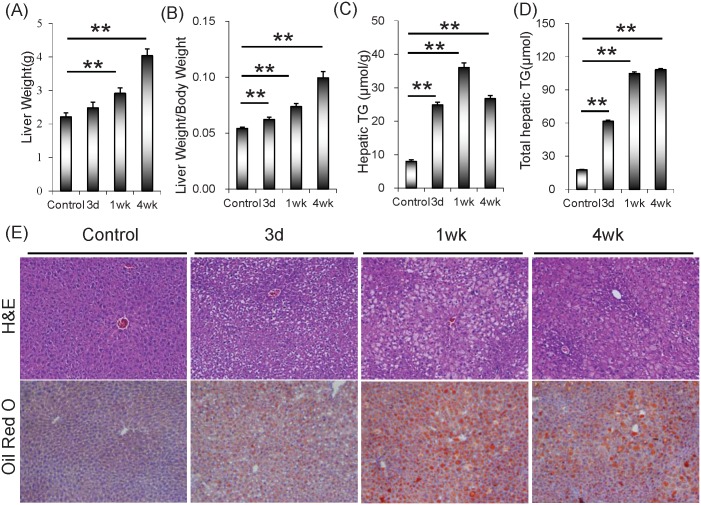
Rifampicin induces hepatic lipid accumulation. All mice except controls were orally administered with rifampicin (200 mg/kg) daily for three days, one week or four weeks, respectively. Liver tissue was collected and weighed. (A) Absolute liver weight; (B) Relative liver weight. (C and D) Hepatic TG content was measured. (E) Hepatic lipid accumulation was evaluated using Oil Red staining. Upper row, representative photomicrograph of H&E staining; Lower row, representative photomicrograph of Oil Red staining. Original magnification, 100×. All data were expressed as means ± S.E.M. (n = 8). ** *P* < 0.01.

Although no significant difference on body weight was observed among different groups (data not shown), the absolute liver weight was significantly increased one week and progressively elevated four weeks after rifampicin treatment (Fig 1A). Correspondingly, relative liver weight was slightly increased three days, significantly increased one week, and persistently elevated four weeks after rifampicin treatment (Fig 1B). The effects of rifampicin on biochemical parameters were analyzed. As expected, serum ALT level was significantly elevated in mice treated with rifampicin (Table 2). Moreover, the levels of serum TG and TG-VLDL were progressively reduced after a short elevation at 3 days after rifampicin treatment (Table 2). In addition, the levels of serum total cholesterol and Chol-HDL were progressively reduced in rifampicin-treated mice (Table 2). The effects of rifampicin on hepatic TG content were then analyzed. In contrast to reduction of serum TG, hepatic TG content was significantly elevated in rifampicin-treated mice (Fig 1C and 1D). An obvious hepatic lipid accumulation, as determined by Oil Red O staining, was observed in rifampicin-treated mice (Fig 1E).

**Table 2:** Serum biochemical parameters.

Parameters	Control	Rifampicin		
		3d	1wk	4wk
Serum ALT(U/L)	23.50±3.09	32.60±3.37**	44.98±6.36**	212.14±55.55**
Serum TG (mmol/L)	1.97±0.13	2.82±0.26**	1.34±0.38*^##^	0.89±0.20**^##^
Serum total cholesterol(mmol/L)	4.40±0.49	3.86±0.40	2.86±0.21**	1.94±0.28**
Serum Chol-HDL(mmol/L)	4.26±0.49	3.40±0.29	2.85±0.21*	1.95±0.26**
Serum TG-VLDL(mmol/L)	0.73±0.05	1.04±0.12*	0.50±0.06**^##^	0.33±0.03**^##^

Data are means ± S.E.M.

* *P* < 0.05,

**P < 0.01 versus control group;

^##^ *P*< 0.01 versus 3d group.

### Rifampicin-induced up-regulation of genes for fatty acid synthesis is independent of hepatic SREBP-1c and LXR-α activation

**Fig 2:**
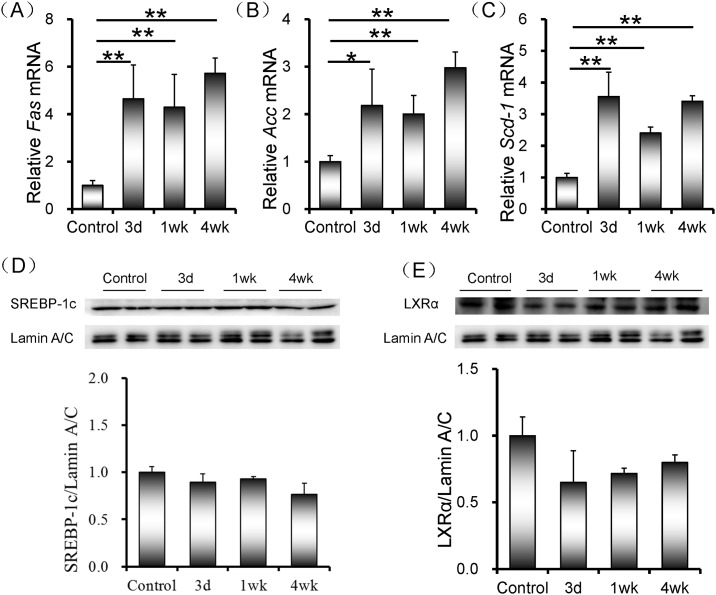
Rifampicin-induced up-regulation of genes for fatty acid synthesis is independent of hepatic SREBP-1c and LXR-α activation. All mice except controls were orally administered with rifampicin (200 mg/kg) daily for three days, one week or four weeks, respectively. Liver tissue was collected. (A-C) Hepatic *Fas*, *Acc* and *Scd-1* were measured using real-time RT-PCR. (A) *Fas*; (B) *Acc*; (C) *Scd-1*. (D and E) Nuclear SREBP-1c and LXR-α were measured using Western blot. SREBP-1c and LXR-α were normalized to the level of Lamin A/C in the same sample. (D) SREBP-1c; (E) LXR-α. All data were expressed as means ± S.E.M. (n = 8). * *P* < 0.05, ** *P* < 0.01.

The effects of rifampicin on the expression of genes for fatty acid synthesis were analyzed. As shown in Fig 2A and 2B, mRNA levels of hepatic *Fas* and *Acc* were significantly increased when mice were administered with rifampicin. In addition, mRNA level of hepatic *Scd-1* was rapidly elevated in rifampicin-treated mice (Fig 2C). SREBP-1c is one of the most important factors that regulate genes involved in hepatic fatty acid synthesis at the transcriptional level. The effects of rifampicin on hepatic nuclear SREBP-1c translocation were analyzed. As shown in Fig 2D, there was no significant difference on the level of hepatic nuclear SREBP-1c between rifampicin-treated mice and controls. LXR-α is another important transcriptional factor that regulates genes for fatty acids synthesis. The effects of rifampicin on hepatic nuclear LXR-α translocation were then analyzed. As shown in Fig 2E, rifampicin had little effect on hepatic nuclear LXR-α level.

### Rifampicin up-regulates expression of genes for ω-oxidation of hepatic fatty acids

**Fig 3:**
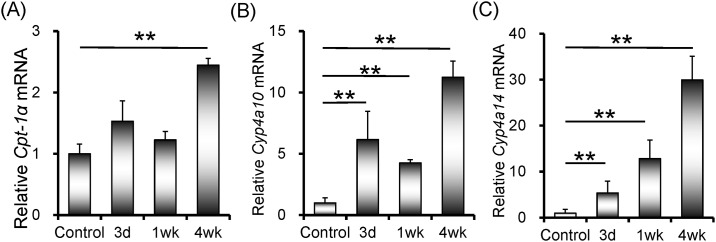
Rifampicin up-regulates expression of genes for ω-oxidation of hepatic fatty acids. All mice except controls were orally administered with rifampicin (200 mg/kg) daily for three days, one week or four weeks, respectively. Liver tissue was collected and hepatic *Cpt-1α*, *Cyp4a10* and *Cyp4a14* were measured using real-time RT-PCR. (A) *Cpt-1α*; (B) *Cyp4a10*; (C) *Cyp4a14*. All data were expressed as means ± S.E.M. (n = 8). * *P* < 0.05, ** *P* < 0.01.

Carnitine palmitoytransferase 1α (CPT-1α) is the key enzyme for β-oxidation of hepatic long-chain fatty acid. The effects of rifampicin on hepatic *Cpt-1α* expression were analyzed. As shown in Fig 3A, mRNA level of hepatic *Cpt-1α* was slightly elevated only in mice treated with rifampicin for four weeks. CYP4A10 and CYP4A14 are two key enzymes for ω-oxidation of hepatic fatty acids. The effects of rifampicin on the expression of hepatic *Cyp4a10* and *Cyp4a14* were then analyzed. Interestingly, hepatic *Cyp4a10 mRNA* was rapidly elevated when mice were administered with rifampicin (Fig 3B). In addition, hepatic *Cyp4a14 mRNA* was progressively up-regulated in rifampicin-treated mice (Fig 3C).

### Rifampicin up-regulates expression of genes for transport of hepatic fatty acids

**Fig 4:**
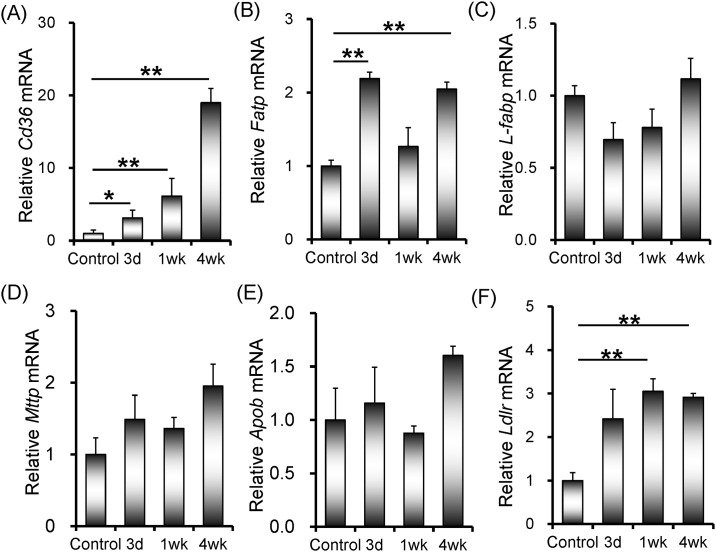
Rifampicin up-regulates expression of genes for transport of hepatic fatty acids. All mice except controls were orally administered with rifampicin (200 mg/kg) daily for three days, one week or four weeks, respectively. Liver tissue was collected. Hepatic *Cd36*, *Fatp*, *L-fabp*, *Mttp*, *Apob* and *Ldlr* were measured using real-time RT-PCR. (A) *Cd36*; (B) *Fatp*; (C) *L-fabp*; (D) *Mttp*; (E) *Apob*; (F) *Ldlr*. All data were expressed as means ± S.E.M. (n = 8). * *P* < 0.05, ** *P* < 0.01.

The effects of rifampicin on genes for transport of hepatic fatty acids were evaluated. As shown in Fig 4A, mRNA level of hepatic *Cd36* was progressively elevated after mice were administered with rifampicin. Moreover, hepatic *fatty acid transport protein* (*Fatp*) and *low-density lipoprotein receptor* (*Ldlr*) were slightly up-regulated in rifampicin-treated mice (Fig 4B and 4F). As shown in Fig 4C–4E, rifampicin had little effect on the expression of hepatic *L-fabp*, *Mttp* and *Apob*.

### Rifampicin up-regulates hepatic PPARγ expression

**Fig 5:**
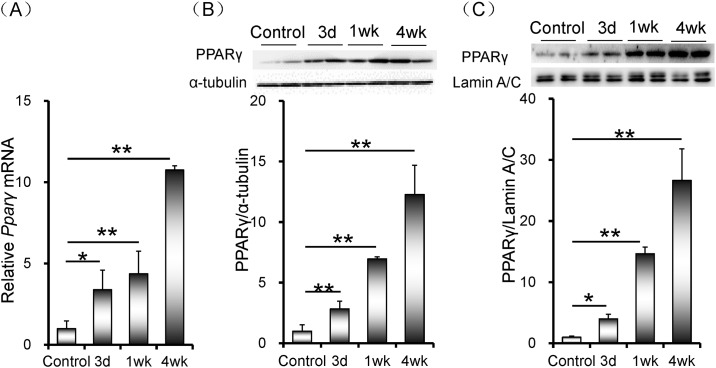
Rifampicin up-regulates hepatic PPARγ expression. All mice except controls were orally administered with rifampicin (200 mg/kg) daily for three days, one week or four weeks, respectively. Liver tissue was collected. (A) Hepatic *Pparγ* mRNA was measured using real-time RT-PCR. (B) Hepatic PPARγ was measured using Western blot. PPARγ was normalized to the level of β-actin in the same sample. (C) Nuclear PPARγ was measured using Western blot. PPARγ was normalized to the level of Lamin A/C in the same sample. All data were expressed as means ± S.E.M. (n = 8). * *P* < 0.05, ** *P* < 0.01.

Peroxisome proliferator-activated receptor γ (PPARγ) is a ligand-activated transcriptional factor. The effects of rifampicin on hepatic PPARγ expression were analyzed. As shown in Fig 5A, hepatic *Pparγ* mRNA was progressively up-regulated when mice were administered with rifampicin. Moreover, the level of hepatic PPARγ protein was markedly elevated in rifampicin-treated mice (Fig 5B). In addition, the level of hepatic nuclear PPARγ was progressively increased in rifampincin-treated mice (Fig 5C).

### Rifampicin activates hepatic PXR signaling

**Fig 6:**
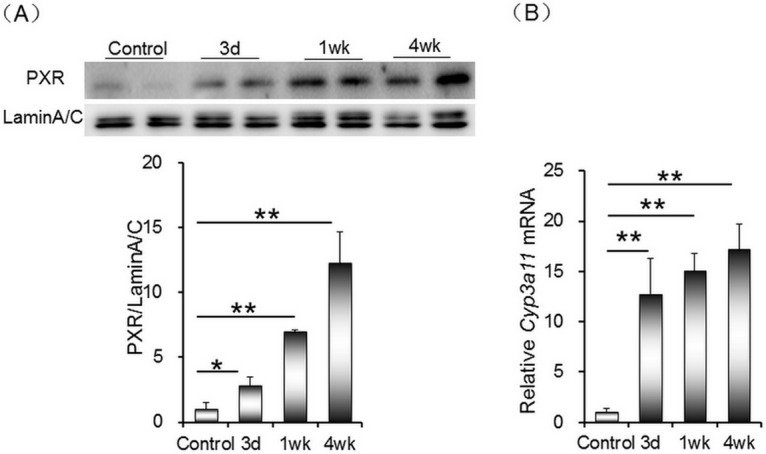
Rifampicin activates hepatic PXR signaling. All mice except controls were orally administered with rifampicin (200 mg/kg) daily for three days, one week or four weeks, respectively. Liver tissue was collected. (A) Nuclear PXR was measured using Western blot. PXR was normalized to the level of Lamin A/C in the same sample. (B) Hepatic *Cyp3a11* mRNA was measured using real-time RT-PCR. All data were expressed as means ± S.E.M. (n = 8). * *P* < 0.05, ** *P* < 0.01.

PXR, which is highly expressed in liver, plays an important role in drug metabolism. The effects of rifampicin on hepatic PXR signaling were analyzed. As shown in Fig 6A, the level of hepatic nuclear PXR was progressively increased when mice were administered with rifampicin. In parallel, mRNA level of hepatic *Cyp3a11*, a downstream target gene of PXR, was up-regulated in rifampicin-treated mice (Fig 6B).

## Discussion

Our previous study demonstrated that rifampicin, one of the most commonly used anti-tubercular drugs, caused intrahepatic cholestasis [15]. The present study investigated the effects of rifampicin on hepatic lipid metabolism in mice. Our results showed that serum TG and total cholesterol levels were progressively reduced when mice were administered with rifampicin for more than one week. By contrast, hepatic TG content was significantly elevated in rifampicin-treated mice. Moreover, an obvious hepatic lipid accumulation, as determined by Oil Red O staining, was observed when mice were administered with rifampicin for more than one week. These results suggest that rifampicin not only impairs bile transport but also hepatic lipid metabolism.

Increasing evidence demonstrates that hepatic de novo lipogenesis contributes much to the development of steatosis [18]. The present study investigated the effects of rifampicin on several key genes for hepatic fatty acid synthesis. As expected, mRNA levels of hepatic *Acc* and *Fas* were significantly elevated when mice were administered with rifampicin for three days. In addition, hepatic *Scd-1* was rapidly up-regulated by rifampicin. These results are in agreement with an in vitro report, in which SCD-1 and long chain free fatty acid elongase were up-regulated in rifampicin-treated HepG2 cells [19]. These results suggest that rifampicin-induced hepatic lipid accumulation is partially attributed to the increased hepatic lipid synthesis.

SREBP-1c is the most important transcription factor that regulates genes for hepatic fatty acid and TG synthesis [20, 21]. The present study investigated whether rifampicin activates SREBP-1c in mouse liver. Our results showed that there was no significant difference on hepatic nuclear SREBP-1c level between rifampicin-treated mice and controls. LXR-α is a nuclear receptor that not only transcriptionally regulates hepatic SREBP-1c and its target genes but also directly modulates genes for hepatic fatty acid synthesis [22–24]. The present study found that no significant difference on hepatic nuclear LXR-α level was observed between rifampicin-treated mice and controls. These results suggest that rifampicin up-regulates key genes for hepatic fatty acid synthesis and elevates hepatic lipid synthesis independent of SREBP-1c and LXR-α activation. PXR is well known as a nuclear receptor that regulates xenobiotic and drug metabolism and elimination [25]. According to an in vitro report, xenobiotic or drug-activated PXR up-regulates FAS expression and promotes de novo lipogenesis via activation of the nonclassical S14 pathway in human hepatocytes [26]. Indeed, the present study showed that hepatic PXR was rapidly activated in rifampicin-treated mice. Therefore, it is reasonable to assume that rifampicin-activated PXR is involved in up-regulation of genes for fatty acid synthesis.

In addition to hepatic de novo lipogenesis, the increased uptake of free fatty acids from circulation to liver plays an important role in the development of hepatic lipid accumulation [27]. The class B scavenger receptor CD36 is a membrane receptor that participates in the uptake of fatty acids from circulation to hepatocyte [28, 29]. Several studies demonstrate that an increased hepatic CD36 expression is associated with enhanced susceptibility to nonalcoholic fatty liver disease [30–32]. The present study investigated the effects of rifampicin on genes for transport of hepatic fatty acids. We showed that hepatic *Cd36* expression was progressively up-regulated after mice were administered with rifampicin. In addition, mRNA levels of hepatic *Fatp*, a fatty acid transporter that participates in the uptake of long-chain fatty acids [33], and *Ldlr*, a surface receptor that removes cholesterol-carrying LDL from plasma by receptor-mediated endocytosis [34], were slightly increased in rifampicin-treated mice. These results indicate that rifampicin-evoked hepatic lipid accumulation is, at least partially, due to the increased uptake of free fatty acids from circulation to liver.

PPARγ is a ligand-activated transcriptional factor that is predominantly expressed in adipose tissues and to a lesser extent in liver [35, 36]. Several studies found that hepatic PPARγ was up-regulated in high-fat diet-fed rodent animals [36, 37]. According to a recent report, PPARγ promotes hepatic lipid uptake and lipid droplet accumulation [38]. Indeed, the class B scavenger receptor CD36 is a downstream target gene of PPARγ [39]. The present study investigated whether hepatic PPARγ was activated by rifampicin. Consistent with hepatic *Cd36* up-regulation, nuclear PPARγ level was progressively elevated in rifampicin-treated mice. The mechanism by which rifampicin activates hepatic PPARγ remains obscure. The present study showed that hepatic *Pparγ* mRNA was up-regulated by more than ten folds when mice were treated with rifampicin for four weeks. Correspondingly, the level of hepatic PPARγ protein was elevated by more than ten folds in rifampicin-treated mice. These results suggest that rifampicin up-regulates hepatic PPARγ expression at a transcriptional level. According to an earlier study, PPARγ is a downstream target of PXR in mouse liver [40]. The present study showed that the level of nuclear PXR was significantly increased by more than ten folds when mice were treated with rifampicin for four weeks. Moreover, mRNA level of *Cyp3a11*, a downstrean target gene of PXR, was significantly up-regulated in rifampicin-treated mice. Taken together, these results suggest that the increased uptake of fatty acids from circulation into liver might be partially attributed to rifampicin-induced PXR activation and PPARγ up-regulation in mouse liver.

CPT-1α is the key enzyme for β-oxidation of hepatic long-chain fatty acid [41]. On the other hand, CYP4A10 and CYP4A14 are two key enzymes for ω-oxidation of hepatic fatty acids [42]. Increasing evidence demonstrates that decreased hepatic fatty acids oxidation partially contributed to the development of NAFLD [43, 44]. The present study analyzed the effects of rifampicin on hepatic β-oxidation and ω-oxidation of hepatic fatty acids in mice. Unexpectedly, hepatic *Cpt-1α* expression was slightly elevated after mice were administered with rifampicin for four weeks. Moreover, the level of hepatic *Cyp4a10 mRNA* was rapidly increased in rifampicin-treated mice. In addition, hepatic *Cyp4a14* was progressively up-regulated by rifampicin. These results suggest that rifampicin-induced hepatic lipid accumulation is independent of the decreased hepatic fatty acid oxidation.

In summary, the present study investigated the effects of rifampicin on hepatic lipid metabolism. Our results showed that rifampicin significantly elevated hepatic TG content and caused hepatic lipid accumulation in mice. We found that rifampicin rapidly elevated expression of genes for de novo lipogenesis and progressively up-regulated genes for uptake of fatty acids in mouse liver. Our results suggest that the increased hepatic lipid synthesis and uptake of fatty acids from circulation into liver jointly contribute to rifampicin-induced hepatic lipid accumulation. The increased uptake of fatty acids from circulation into liver might be partially attributed to rifampicin-induced up-regulation of PPARγ and its target genes.
